# Recent Studies on Supercapacitors with Next-Generation Structures

**DOI:** 10.3390/mi11121125

**Published:** 2020-12-18

**Authors:** Juho Sung, Changhwan Shin

**Affiliations:** Department of Electrical and Computer Engineering, Sungkyunkwan University, Suwon 16419, Korea; jhsung0606@gmail.com

**Keywords:** supercapacitor, electrical double layer capacitor, EDLC, negative capacitance, energy storage system, pseudocapacitor, micropores, nanopores, ultracapacitor, Helmholtz model

## Abstract

Supercapacitors have shown great potential as a possible solution to the increasing global demand for next-generation energy storage systems. Charge repositioning is based on physical or chemical mechanisms. There are three types of supercapacitors—the electrochemical double layer, the pseudocapacitor, and a hybrid of both. Each type is further subdivided according to the material used. Herein, a detailed overview of the working mechanism as well as a new method for capacitance enhancement are presented.

## 1. Introduction

The Fourth Industrial Revolution, which facilitates the emergence of a hyper-connected society, is the basis for data processing between people and things as well as things and things. The essence anchored in a host of electronic devices highlights the energy consumption problem to address the ever-increasing energy demand. However, the challenge confronted (based on expenditure) is proving hard to overcome. Self-powered devices are mostly based on lithium-ion batteries, which were proposed three decades ago. They demonstrate superior properties as compared to energy storage systems (ESSs) based on other materials with the highest energy storage capacity; however, the world no longer refers to them as “perfect batteries”. Moreover, the progress of battery technology would be considered as a stumbling block to the development of electrically powered industry in some parts of the world. Insufficient cycles to deliver energy owing to membrane shrinkage by shutdown, power density, and thermal stability are now more noteworthy aspects in particular fields. Most importantly, the energy storage capacity, which is considered a strength, is no longer increasing.

The major frameworks to solve these issues are classified as follows: (1) ultra-low-power devices based on energy efficiency with various techniques to overcome the theoretical limit of energy consumption efficiency with constant energy capability [1,2,3,4] and (2) focusing on developing the next generation of ESSs to complement lithium-ion batteries that have reached their innovation limit in a specific area [5,6,7].

There are various requirements to implement these next-generation ESSs, but to date some systems to satisfy certain conditions remain nonexistent [8]. Of these, capacitors using the extreme surface area and distance from the electrode to the charged ions layer on the electrolyte have particularly attracted attention owing to their superior specific power. Capacitors with superior characteristics (called supercapacitors), which are unavailable in conventional batteries, exhibit excellent functionality in many areas, including power density, charge/discharge cycles, operation over a wide temperature range, and reliability, which have been noted as limitations of batteries [8,9,10,11,12,13]. However, based on energy density, it is evident that supercapacitors must be studied intrinsically.

Among a few solutions for overcoming the limitations of low energy density, the main area of interest involves the development of new materials or structures for the electrodes of supercapacitors. The electrode materials in laboratories and industries are typically reliant on carbon, metal oxides, mixed metal oxides, and conductive polymers. Since the onset of supercapacitor manufacturing, carbon materials have been used because of their broad surface area [7,14,15]. Metal oxides are mainly used for pseudocapacitors, and due to their high capacity and low resistance they can be attractive as electrode materials which have a higher energy and power than conventional carbon-based supercapacitors. For conductive polymers, a reduction-oxidation process is used to store and release charges. Various conductive polymers have been widely studied because they are easy to produce and are inexpensive compared to other materials fabricated for supercapacitor electrodes [11].

Recently, a new theory was proposed with a layer combination of a ferroelectric exhibiting negative capacitance and dielectric, which could enhance the capacitance that had been hitherto considered as an inherent limitation. In addition to previously exhibiting the ability to handle low-power technologies [1,2,3,4], ferroelectric materials with negative capacitance effects have demonstrated the ability of ESSs in combination with dielectric layers. The theory is based on ionic polarization, which leads to ferroelectric effects, as well as dipolar polarization. It has evinced an improved capacitance, which resulted in a theoretical increase in storage energy of up to two times and also experimentally achieved an increase in storage energy of up to 1.5 times [16]. This presents a new possibility to increase capacitance which can overcome the limitations of supercapacitors. The results were experimentally confirmed, as comprehensively described in Section 4.

This article not only describes the analysis of supercapacitors based on various electrochemical double-layer models but also introduces new ways to improve capacitance beyond the fatal limit by utilizing negative capacitance materials.

The characteristics of capacitors, supercapacitors, and batteries, which are typical ESSs, are described in Table 1. The indicators for specific energy density, specific power density, charge storage mechanism, cycling performance storage region, charge temperature, discharge temperature, and galvanostatic discharge curves are presented. In the case of galvanostatic discharge curves, this allows us to figure out that the capacitor and supercapacitor could use the total stored energy rapidly through the slope of curves. In contrast, the battery shows a relatively uniform state.

## 2. Classification of Supercapacitors

**Figure 1:**
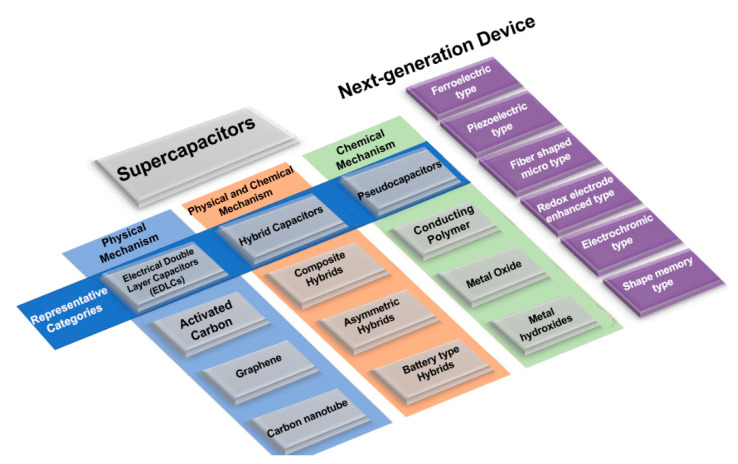
Classification of supercapacitors [24].

Supercapacitors, which are also referred to as double-layer capacitors, gold capacitors, power caches, power capacitors, ultracapacitors, or super condensers, are classified into three types, depending on the charge storage mechanism: (1) the electrical double-layer capacitor (EDLC), which is based on a charge storage mechanism that physically stores charges on the electrode surface with an electrical double layer without causing irreversible chemical reactions [8,9,10,11,17]; (2) a pseudocapacitor, which is operated by a chemical reaction that relies on a mechanism that stores charge only in a limited area based on surface, unlike a battery, wherein the reaction occurs on the spacious electrode region from the surface to the bulk region; and (3) hybrid capacitors that adopt both mechanisms, composed of EDLC electrodes and electrodes based on chemical mechanisms, such as pseudocapacitors or batteries, which can exhibit the characteristics of both systems and provide intermediate performances. Here again, they are subdivided according to the material of the electrode or electrolyte (see Figure 1) [18].

Notably, EDLCs can withstand millions of cycles owing to the mechanism that stores charge on the electrode surface with an electrical double layer based on a constantly reversible capability. This property allows the device to not only exceed the life of the system but also the quality of the device compared to batteries. The reversible capability does not stir deviation up in the electrode, thereby eliminating the swelling that occurs in typical redox reactions found in spacious amounts of battery active materials during charge and discharge cycles. Supercapacitor electrodes undergo electrochemical dynamics through polarization resistance, and no rate limiting occurs within the bulk range as in redox battery electrodes. Based on the EDLC structure, many research teams have spent their efforts on improving the charge storage quality regarding the high specific surface area of porous materials, which are mainly used for electrodes.

Until pseudocapacitors based on intercalation were embraced, pseudocapacitors have also been synonymously used with “surface redox reactions”. Redox-active materials, such as metal oxides, mixed metal oxides, sulfides, selenides, nitrides, conductive polymers, carbon materials, and composite with carbon materials, have been used on the active electrode. The energy based on the chemical reaction of a solid substance is mainly stored in chemical bonds—that is, ionic bonds, covalent bonds, and hydrogen bonds. When exploited in electrochemical reactions, solid materials with high bond energies are converted into low bond energy materials to release electricity. Electrical and chemical potentials in the electrolyte can be reversibly converted by an interaction between charged/discharged electrode materials and chemical reactions. Pseudocapacitors do not depend on metal chemistry in the bulk area; thus, there is less risk of metal plating, which poses significant performance degradation, failure mechanism, and safety issues that can lead to short-circuiting and uncontrollable energy chemical reactions [6,18,19,20].

The material conventionally used in the active region of the electrode is described in Table 2. Active carbon, carbon nanotube(CNT)/graphene, metal oxide pseudocapacitance, metal oxide faradaic reactions, and conducting polymers are described according to their benefits, issues, and electrode descriptions [21,22].

Pseudocapacitors based on redox reactions as well as other chemicals exhibit higher specific capacitance properties than EDLCs based on physical charge storage operations. However, there are advantages and disadvantages of energy storage via physical and bulk chemical reactions. 

The concept of exploiting both physical and chemical mechanisms is presented as hybrid supercapacitors. This is a combination of materials commonly used in pseudocapacitor-based electrodes–electrolytes with EDLC-based electrodes and lithium electrode–electrolytes with EDLC -based electrodes. The second approach is considered as a lithium-ion capacitor, which mainly entails the use of metal oxides or mixed metal oxides and carbon electrodes to construct the cell. Owing to this architecture of arrangement, the storage cell functions in a wider working potential window, thereby exhibiting a larger capacitance value. This approach provides a 2–3-times higher energy density than that of traditional EDLCs [21].

## 3. Electrical Double-Layer Capacitors (EDLCs)

### 3.1. Theory of the Electrochemical Double Layer

As the name itself indicates, supercapacitors are devices that maximize the capacitance of conventional capacitors. These devices based on physical charge storage with electrical operation exhibit a charge output like capacitors; thus, the capacitance “*C*” depends on the dielectric constant *ε_r_* of the electrolyte, the effective thickness “*d*” of the charge separation layer from electrodes, and the accessible surface “*A*” through the distance between the electric layers based on Helmholtz theory and the porous structure [21].

In conventional capacitors, surface area A is a two-dimensional single-planar electrode, while the supercapacitor is a three-dimensional porous electrode—i.e., the surface area is maximized. The dielectric layer of a supercapacitor comprises a single or several molecular layers, so “*d*” is only a small molecular distance, ranging from a few Å to several Å. This is a huge difference, considering that an electrostatic capacitor has an insulator layer that reaches up to several microns.

**Figure 2:**
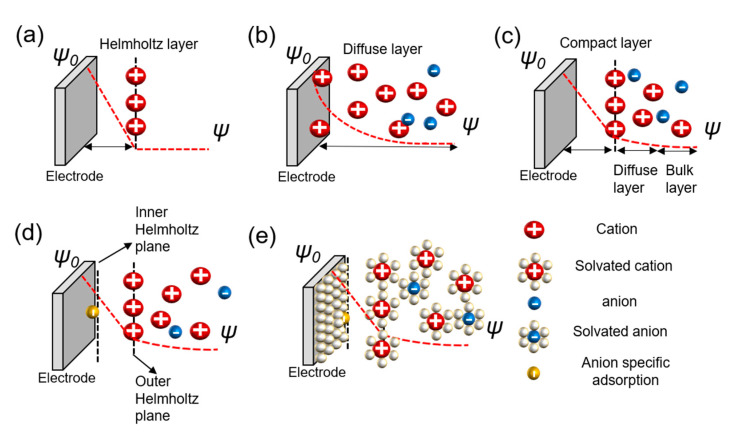
Models of the electrochemical double layer. (**a**) Helmholtz model; (**b**) Gouy–Chapman or diffuse model; (**c**) Stern model; (**d**) Grahame model; (**e**) Bockris, Devanathan, and Muller model [25,26,27].

The charge separation layer is formed with ions when a charged object is placed in an electrolyte. The mutual interfacial structure between the electrodes creates a certain structure. The balancing counter charge on the charged surface of this object is formed correspondingly in the electrode and concentrated near the surface. Various theories have been proposed to explain the structure wherein charges are formed, and a complete theory explaining all the mechanical contents remains undetermined. There are theories for understanding the charge separation layer, as represented in Figure 2. 

The Helmholtz model is the first theory to provide an approximation for the arrangement between the electrode and layer of ions called the Helmholtz layer or plane with a finite distance. However, there are some problems with interactions that occur further away from the electrode which include the electrolyte concentration and all the given environments, besides the double layer arising from the interface (Figure 2a) [25,28]. Chapman did not take this into account and proposed an electrical double-layer theory and considered the applied potential and electrolyte concentration. Gouy suggested that a layer of positive ionic charge that counteracts the charge affecting the charged electrode appears in the electrolyte surrounding the charged solid, but that ions did not appear only on the surface with a diffusion layer based on the Boltzmann distribution that demonstrated the concentration of charged ions with the counter ions near the surface. This is called the diffuse layer. However, this model also has problems. Because ions are expressed as point charges, the specific size of the ion is not considered. The theory does not recognize that the finite space of ions cannot exist near the electrode. Secondly, it fails when applied to highly charged double layers (Figure 2b) [25,29,30,31,32,33,34]. Stern modified the diffusion double layer to integrate the theories of Helmholtz and Gouy–Chapman and proposed the compact layer presented by the improved understanding of the architectures of the ions with a specific size and limiting space between the electrode and electrolyte, but also extended the diffusion layer to the bulk layer. This distance is generally considered as the radius of the ions. Consequently, the potential charges and concentration of the diffusion portion of the layer are sufficiently low to justify treating ions with point charges (Figure 2c) [25,35,36,37]. Grahame developed the theory of electrical double layers based on four regions—the inner Helmholtz plane in front of the electrode with small molecular distances; the outer Helmholtz plane, which consists of Helmholtz layers; the diffusion region; and the bulk region suggested by Stern. The theory of specific adsorption was applied. This bond occurs on the surface of the metal electrode. The negative ions are adsorbed on the surface of the electrode regardless of its charge, not by the electrical force, but via chemical interaction. The ions referred to as ghost ions act as pseudocapacitive particles. Cations rarely stick to the electrode. The adsorption of ghost ions would be reduced while keeping the solution and electrodes clean. The capacitor centered on the reaction by specifically adsorbed ions is called a pseudocapacitor, which is found at the interface between the electrolyte and electrode (Figure 2d) [25,26,27]. Bockris, Devanathan, and Muller’s model shows a phenomenon for the preponderance of solvent molecules around the interface. Solvated molecules, anions, cations, and electrodes were suggested, and the dipole moment was considered for both the charge separation and diffusion layers. The model is acceptable for the values applied to understand high-current-power EDLCs. However, the concept cannot perfectly explain carbon-based models with porous structures (Figure 2e) [25,38].

### 3.2. Variation of Capacitance According to Pore Size and Structure

Although the theory of electric double layers on plane surfaces has been clarified, problems arise when applying electric double layers to pores in actual spaces. The characteristics of ion electrical adsorption in porous structures make the process of charge storage difficult to deal with. Understanding the principle of charge storage through the formation of layers by cations and anions remains insufficient to explain experimental results when applied in nanopores. Generally, the inverse effect of pore size on capacitance is also known. Ions have different movement patterns with pores compared to the surface or bulk region. 

The specific surface area, which exhibits an evincive connection between the pore sizes, is important in order to understand its impact on specific capacitance. Additionally, it has been an attractive subject of study to numerous research groups in the past two decades. The capacitance generally increases proportionally to the surface area, but in the micropore region a difference in increasing value occurs. As the movement of ions has architectures with a finite size, the potential charges are greatly affected by the size of the pores. Each ion that plays a role in the storage of charge has a specific size, thereby allowing access to structures above a certain scale. Even if the surface area is increased, in regions where the pore size is too small for the solvated ion to approach it does not fully contribute to the layer, which should interact with electrodes. The specific and volumetric capacitance values indicate significant increases and are inversely commensurate to the nonlinear proportion depending on the chlorination temperature, which is largely involved in pore size control [39,40].

It is important to adjust the temperature, density, and intensity values for each material. The targets that constitute the electric double layer with the ions in the electrode layer are activated carbon and graphene, which have two-dimensional structures, and carbon nanotubes (CNTs), which have three-dimensional structures. Activated carbon changes the pore size depending on the temperature. Because the size of the pores determines the number of ions that can pass through the bilayer, if the conductivity is greatest at one point in the nano-meter range, then it decreases rapidly at higher and lower temperatures based on that state [39,40,41,42,43,44].

**Figure 3:**
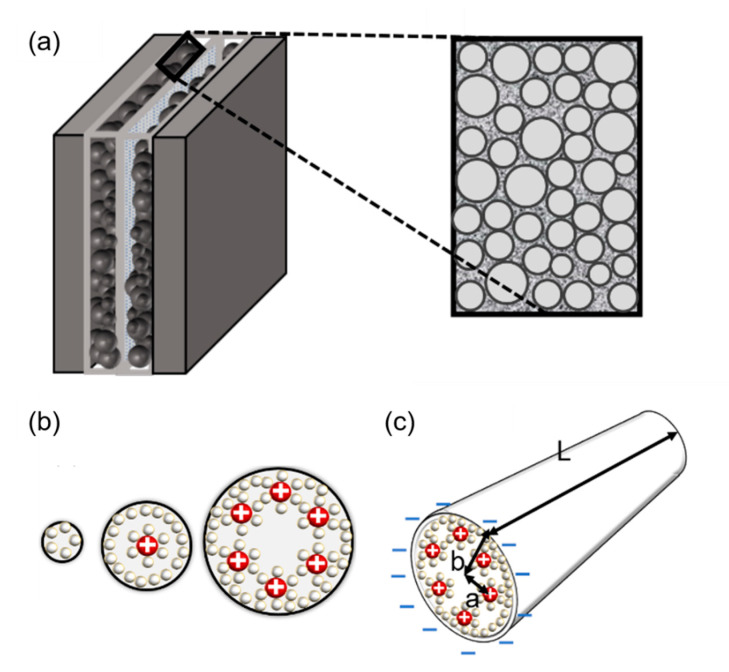
(**a**) Brief structure of a supercapacitor with a view of cylindrical theory (endohedral capacitors/electric double-cylinder capacitor), (**b**) shape of the electrolyte ion placed and the cylindrical electrode according to the pore size. The first picture shows that the ions are not very involved in charge storage because they have a very small pore structure. The second figure shows one ion interacting with an electrode to store charge. The third figure shows more ions forming the same layer and acting on the electrode. (**c**) The shape of pores observed in a three-dimensional shape; “*a*” is the distance from the center to a layer of several ions, the radius of the pores is expressed as “*b*”, and the cylinder length is expressed as “*L*” [21,45,46].

The reasons for this are as follows. First, when they are smaller than the largest point, solvated ions do not have the minimum length to form a double layer. That is, the optimal pore size may vary depending on the scale of the electrolyte ions (Figure 3) [42,43,44]. According to their characteristics, there is a change in the interaction mechanism between the electrodes and ions. To understand this phenomenon, micropores are analyzed from various perspectives. The first aspect concerns layer formation as a cylinder (Figure 3).

There are many inquiries regarding the interaction between the electrolyte and the electrode fabricated with carbon material to adequately describe the electrostatic capacity depending on the pore shape and size. The analysis of capacitance for a cylindrical model is as follows, and it is analyzed by Gauss’s law (Figure 3c):

(1) $$V_{ba}=-\int_{a}^{b}(\frac{Q}{2\pi \varepsilon_{r}\varepsilon_{0}rL})dr$$

(2) $$V_{ba}=-\frac{Q}{2\pi \varepsilon_{r}\varepsilon_{0}L}\int_{a}^{b}\frac{dr}{r}$$

(3) $$V_{ba}=-\frac{Q}{2\pi \varepsilon_{r}\varepsilon_{0}L}\ln\frac{b}{a}.$$

In the equation, “$\varepsilon_{r}$” represents the dielectric constant of the electrolyte, the permittivity of a vacuum is expressed as “$\varepsilon_{0}$”, the distance from the center of the pore to the cation is expressed “*a*”, and the radius of the pore is expressed as “*b*”.

(4) $$Q=CV_{ba},$$

(5) $$C=2\pi \varepsilon_{r}\varepsilon_{0}L{\left(\ln\frac{b}{a}\right)}^{-1},$$

where the total charge on the cylinder is expressed as “*Q*”, and *C* represents the capacitance of the supercapacitor. This is applied at a distance of ~0 nm or less from the electrode to ions. Depending on the size of the pores, the number of ions that can contribute to the capacitance and the size of the layer of ions change (Figure 3b).

**Figure 4:**
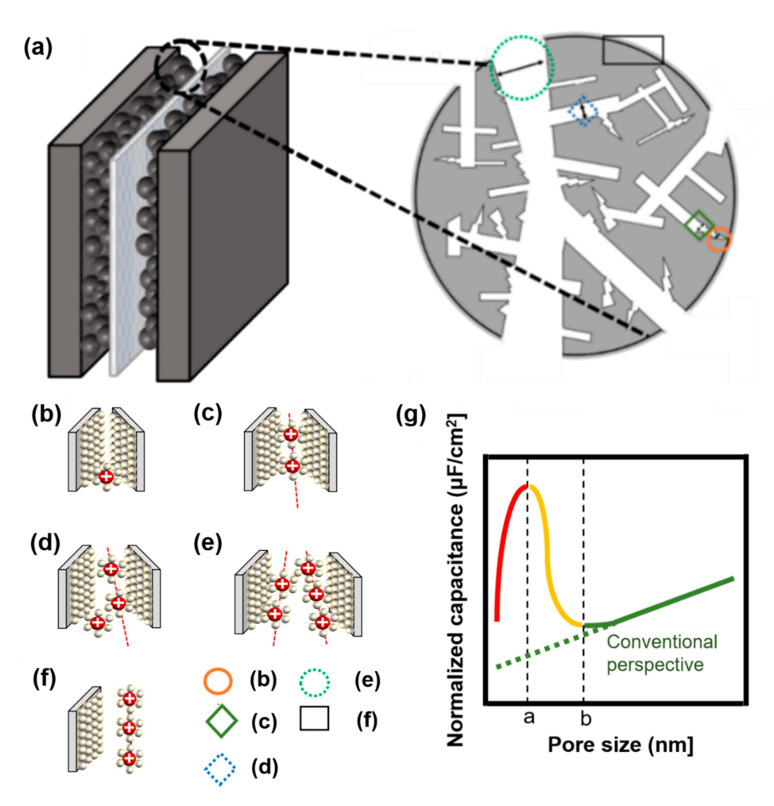
(**a**) Brief structure of activated carbon particles with various pore sizes, not based on a circle, but based on the electrode theory of walls and walls. (**b**) Nanopores do not have the minimum space required to form an electrical layer with solvated ions, (**c**) nanopores have minimal space to form an electrical layer with solvated ions, (**d**) nanopores have extra space to form an electrical layer with solvated ions, (**e**) nanopores have sufficient space for each electrode such that solvated ions can form an electrical layer with each electrode, (**f**) electric layer for carbon grain at the surface, (**g**) schematic diagram of normalized capacitance according to pore size. When the pore size is less than or equal to *a*, which is indicated by a red-colored line, (**b**,**c**) is applicable, and when the pore size is more than *a* and less than or equal to *b*, which is indicated by a yellow-colored line, (**c**,**d**) is applicable. If the pore size is larger than b, which is indicated by a green-colored line with a conventional view, (**e**,**f**) is applied [25,39,40,42].

The second model tries to understand the interaction between ions and electrodes based on slits. Depending on the interval between the slits, the types wherein the ions of the electrolyte with electrode contribute are different which is described on Figure 4b–f [45,46].

If it is larger than point *a* in Figure 4g, the distance between the electrode and ions increases because one electrode and several solvation ions constitute a double layer. Here, solvation ions act as an insulator, and the distance is applied to the capacitor’s width of the dielectric layer. When the pore size is greater than a certain spot *b* in Figure 4g, the electrode pores and the layer of ions that can be formed start to increase; thus, the capacitance starts to increase again [39]. Nanopores sometimes have minimal spaces to form electrical layers with solvated ions (Figure 4b) as well as electrical layers with solvated ions (Figure 4c). If the pore size is less than or equal to *a*, (b) → (c) applies. (d) represents the case of solvated ions with nanopores having extra space to form an electrical layer, and (e) shows that the nanopore has sufficient solvated ions to form an electrical layer with each electrode and that there is adequate space in each electrode. If the pore size exceeds *a* and *b* or less, (c) → (d) is applicable. If the pore size is greater than *b*, (e) → (f) is applied (Figure 4) [39,40,42].

Depending on how the capacitor interacts with the carbon surface of the counter ion, these EDLCs can also be divided into endoderm and ectoderm capacitors, distinguished by the way the carbon surface interacts with the charged molecule layer. In the endoderm, counter ions enter the pores and form EDLs with the external electrodes. This type of charge accumulation is observed for nanoporous carbons with negative surface curvatures. These structures are conventionally shown on the activated carbon, carbide-derived carbon, and template carbon. In the case of an outer surface capacitor, it was observed that ions existed on the outer surface of the carbon particles [21].

**Figure 5:**
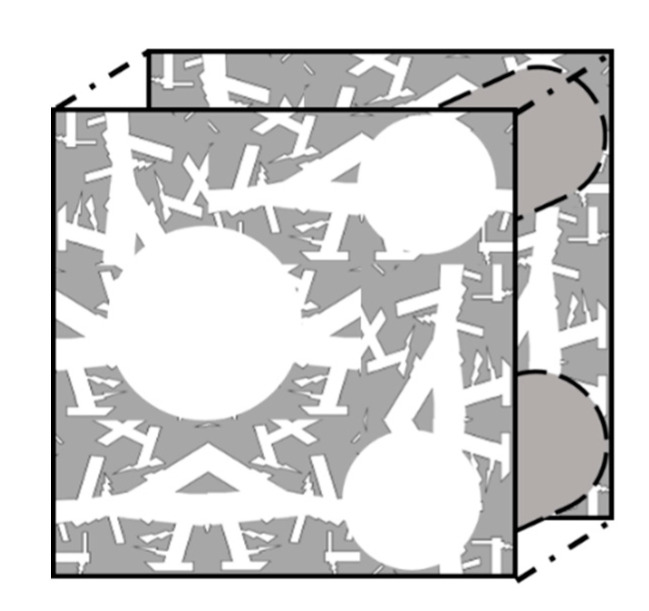
An analysis model for the theory of nanopores, showing a model that combines the theory of a structure made of cylinders and micropores [25,39,40,42,45,48].

**Figure 6:**
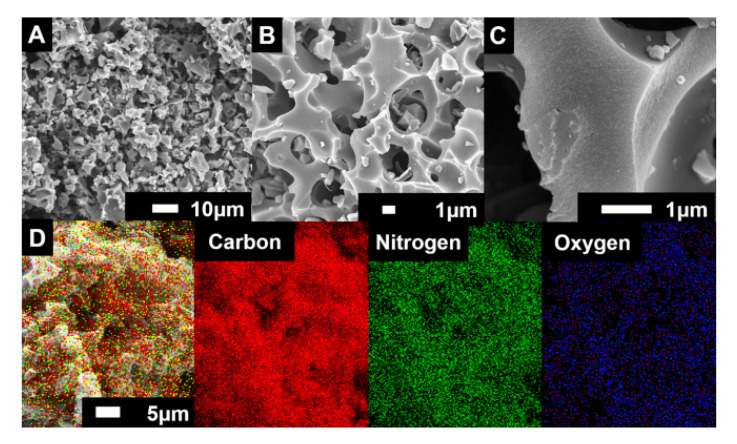
(**A**–**C**) Macro/micropole-dominated carbon on SEM (scanning electron microscope) images with different scale (10 μm and 1 μm) and (**D**) element mapping of highly porous activated carbon [47]. Reprinted with permission from Ref. [47]. Copyright 2017 American Chemical Society.

The third model tries to interpret both theories with a cylindrical and slit structure (Figure 5). This theory suggests that there are significant cylinders that affect the normalized capacitance and slits which are based on micropores with inaccessible architectures according to the size of the pores. As this theory combined conventional theories about micro and macropores, it is more compatible to interpret lots of phenomena. The cylinder radius size plays a role of consolidation with capacitance, since the pore size reaches certain values. Additionally, the size of the slits affects the normalized capacitance, since the size is changed with various ranges [47,48]. Both the macropore and micropore structure is captured by scanning electron microscopy (SEM) images of highly pores activated carbons (HAPC). The images by SEM are 1 μm to 10 μm with different elements (Figure 6) [47].

Carbon materials with positively curved surfaces, such as nanofibers and carbon ions, exhibit certain types of behaviors. Since the curvature of graphene is zero, it does not belong to the first two categories, and it is classified as a graphene capacitor itself.

The capacitance in the plane is shown in series with the electrical double layer, wherein “*a_0_*” is few molecular distances which are radii to solvated cation, the half of distance from the slit is expressed as “*b*”, and “*d_eff_*” is the effective separation distance between the electrode surface and the oppositely charged ions, which do not simply point charges, and the ionic radii place a role of the location of the charge densities. 

**Figure 7:**
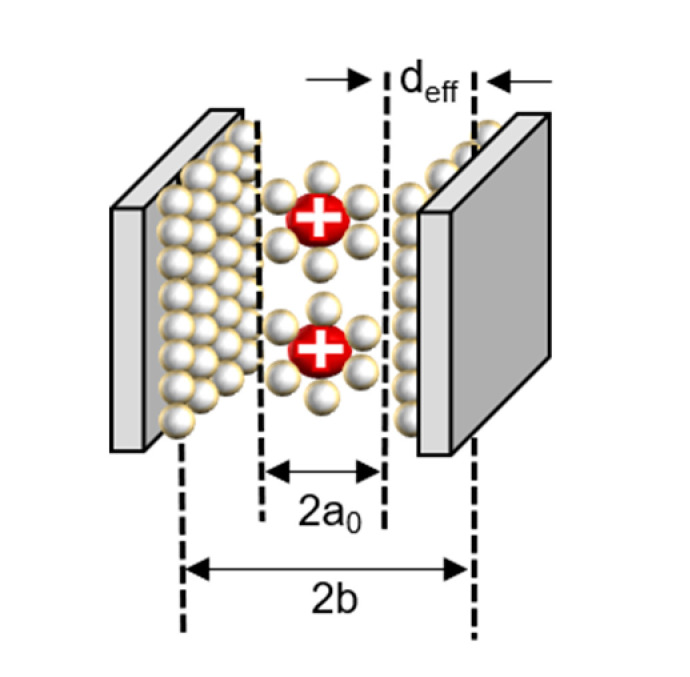
Schematic diagram of a capacitor formed by a solvated cation with a negative polarity located in the middle of two electrodes separated by a single layer of charge [39,44,50].

In the recently discovered carbon, a realistic approximation of the pore shape is a slit rather than a cylinder. Accordingly, a polarity located in-between two electrodes separated by a single layer of charge has been proposed. The capacitive formula for the sandwich capacitor, which is located in between two electrodes of the same polarity and is formed with a layer formed with a counter ion separated with *2b*, which is indicated in the pore width (Figure 7). Because the counter electrode shares the total net charge of the opposite charged ions, slit capacitors can be considered as two capacitors in parallel, represented by the left and right-hand electrodes and the charged molecule layer in the middle, as shown in Figure 7. Therefore, the total capacitance *C_tot_* is calculated as follows [44,49]:

(6) $$C_{tot}=\frac{2\varepsilon_{r}\varepsilon_{0}A}{d_{eff}}.$$

In the equation, $\varepsilon_{r}$ represents the dielectric constant of electrolyte, $\varepsilon_{0}$ is the permittivity of a vacuum, and the surface area of the electrode is represented as *A*.

(7) $$d_{eff}=\left(b-a_{0}\right),$$

where *d_eff_* is the effective separation distance between the electrode surface and the opposite charged ions, and the total capacitance of the capacitor is obtained as follows:

(8) $$C_{tot}=\frac{2\varepsilon_{r}\varepsilon_{0}A}{b-a_{0}}.$$

All the analyses were performed in one region, similar to the interaction method between the dielectric layer and the electrode, which was performed in a typical capacitator. The theory notified that the corrections of charge separation by the locations of charge densities are extremely important in producing a reliable capacitance model, especially for micropores. However, as the present model is derived for parallel plates, we should notice that a precise structure of the electrode was not considered for the reason of feasibility. The areal total capacitance of the system should be obtained [44].

## 4. Negative Capacitance for Capacity Enhancement

A polarized molar solvent is generated in the compact double layer, which acts as an insulator, and becomes polarized to form the electrode surface and maintains dipole planes. Recently, theories and experiments have been proposed involving a combination of negative capacitance and dielectric material, which can enhance the capacitance [16].

**Figure 8:**
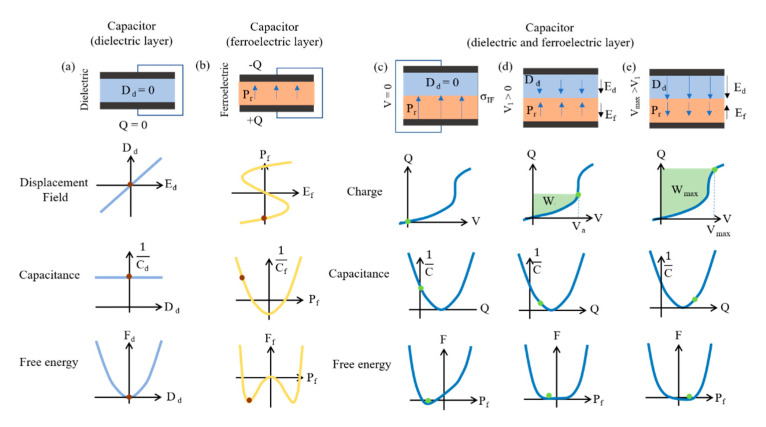
Illustrations and plots to express the integration of capacitance caused by both the dielectric layer and the ferroelectric layer to enhance capacity. (**a**) Schematic of the dielectric capacitor including *D_d_*-*E_d_* curve, *C_d_*^−1^*D_d_* c-urve, and *F_d_*-*D_d_* curve without external bias. (**b**) Schematic of ferroelectric capacitor including *P_f_*-*E_f_* curve, *C_f_*^−1^-*P_f_* curve shown as “s” and *F_f_*-*Pd_f_* curve with polarization on 2Q. (**c**–**e**) Schematic of capacitor compounded with both ferroelectric and dielectric layer shows significant charge storage which is expressed as the area above the Q-V curve. (**c**) shows a state at zero bias, (**d**) demonstrates at *V_a_*, and (**e**) expresses at *V_max_*. Reprinted with permission from Ref. [16]. Copyright 2019. The authors are under Creative Commons License 4.0 (https://creativecommons.org/licenses/by/4.0/).

The electrical properties of a dielectric capacitor could be expressed with *F_d_*, which indicates free energy; *D_d_*, which means displacement field; and *C_d_*^−1^ inverse capacitance without external bias. A capacitor with a ferroelectric layer is also expressed with its corresponding parameters with *F_f_*, *C_f_*^−1^ and *P_f_* mean spontaneous polarization. To fabricate supercapacitors using negative capacitance, supercapacitors should be consolidated with a ferroelectric layer on the dielectric stack. Secondly, since the depolarization field on the ferroelectric layer could affect the spontaneous polarization state, interfacial charge, which is expressed with *σ_IF_*, should be fabricated between ferroelectric and dielectric layers to adjust *P_f_* at zero voltage. Ferroelectric-dielectric capacitor could enhance its capacity with the integration of electrical energy by polarization on the ferroelectric layer. As the ferroelectric layer based on negative capacitance could demonstrate a significantly high ratio, the capacity is abruptly enhanced at *V_a_*. These fabrication processes are interpreted in Figure 8. 

The improvement in capacitance resulted in an increase in the storage energy up to 1.5 times. Ionic polarization induces ferroelectric effects as well as dipolar polarization. Ferroelectrics that lead to negative capacitance may be applied in the electrolyte, and ferroelectric materials may be deposited on the active carbon layer to establish the effect of negative capacitance.

The insulator layer formed through oxidation on the general carbon layer played a role in inducing capacitance reduction. This is the reason why the distance increased as the insulator layer was formed in the molecular dielectric layer, and the insulator layer formed through oxidation has a low dielectric constant. However, ferroelectrics exhibited increased capacitances when they encountered the dielectric layer and had a high dielectric constant. The theory for the enhancement of capacitance with a combination of the dielectric and ferroelectric layers is given by:

(9) $$V_{d}=\frac{Q}{c}.$$

*V_d_* is the voltage applied to the dielectric capacitor.

(10) $$V_{f}=a\left(Q-\Delta Q\right)+b{\left(Q-\Delta Q\right)}^{3}.$$

*V_f_* is the voltage applied to the ferroelectric capacitor.

(11) $$V_{tot}=\left(\frac{1}{c}+a\right)Q-a\Delta Q+b{\left(Q-\Delta Q\right)}^{3}.$$

*V_tot_* is the voltage applied to the dielectric–ferroelectric capacitor.

The total energy value for a capacitor combined with a combination layer is summarized as follows:

(12) $$W_{tot}=\int_{0}^{Q}V_{tot}\left(Q\right)dQ.$$

The equation for energy is given by:

(13) $$W_{tot}=\left(\frac{1}{2c}-a\right)Q^{2}-b\Delta QQ^{3}+\frac{b}{4}Q^{4}.$$

*W_tot_* is the total energy stored on the dielectric–ferroelectric capacitor. After normalizing *W*, the difference with the existing capacitance should be summarized to the whole storage system at the same voltage to use the steep slope region of the ferroelectric capacitor. Using the first-order approximation on the $w_{0}=CV^{2}/2$ by Taylor’s expansion terms yields the following:

(14) $${\frac{W}{w_{0}}|}_{v=v_{max}}≅1-aC.$$

The equation of normalizing *W* could explain the independence of b and rely only on the product “*aC*”. When the product is equal to the value of “−1” for optimizing the capacitance matching, the enhancement of *W* becomes approximately twice as great, meaning that the energy stored at the identical voltage is doubled [16,51]. The experimental results according to the theory did not show a significant difference and indicated an increase in energy storage of up to 1.5 times. However, the experimental result was only applicable to solid dielectric layers, and not to electrolytes; thus, it can be expected to have different effects when applied to supercapacitors using molecular solvent on electrolytes as the dielectric layer.

**Figure 9:**
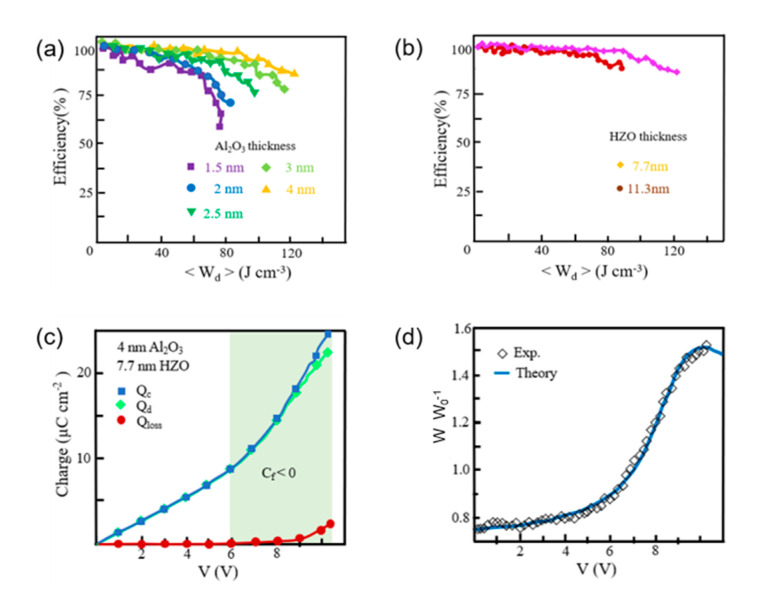
Plots of capacitor compounded with both a ferroelectric (HZO) and dielectric layer (Al_2_O_3_) with various layer thicknesses. (**a**) Efficiency (%) versus discharged energy density 〈*W_d_*〉(J/cm^−3^) for 7.7 nm HZO compounded with 1.5 nm to 4 nm Al_2_O_3_ layer thicknesses. (**b**) Efficiency (%) versus discharged energy density 〈*W_d_*〉(J/cm^−3^) for 4 nm Al_2_O_3_ compounded with 7.7 nm and 11.3 nm HZO thicknesses. (**c**) Charging (*Q_c_*), Discharging (*Q_d_*), and Charge loss (*Q_loss_*), which means *Q_c_*-*Q_d_*, according to measured charges versus applied voltage at capacitor fabricated with the 4 nm Al_2_O_3_/7.7 nm HZO. (**d**) Charges versus applied voltage with an experimental and theoretical capacity increase (*W*/*W*_0_). Reprinted with permission from Ref. [16]. Copyright 2019. The authors are under Creative Commons License 4.0 (https://creativecommons.org/licenses/by/4.0/).

Although theoretical efficiency is expressed as 2 times that of the conventional device, the capacitor with HZO (ferroelectric) layer and Ta_2_O_5_ (dielectric) layer (to match *aC*) has exhibited the enhancement (*W*/*W*_0_) about 1.1 due to the value of *aC* which is exhibited about −0.16. The thickness of both HZO and the Al_2_O_3_ layer is investigated about the effect on the energy storage properties. Figure 9 depicts efficiency (%) versus discharged energy density 〈*W_d_*〉 (J/cm^−3^) curves for 7.7 nm HZO thickness with various Al_2_O_3_ thicknesses. Increasement of Al_2_O_3_ thickness which means volume fraction of the dielectric material affects not only the increase in the maximum obtainable 〈*W_d_*〉 but also efficiency. The maximum obtainable 〈*W_d_*〉 is achieved for 7.7 nm HZO and 4 nm Al_2_O_3_ with 121 J cm^−3^. The ratio of dielectric/ferroelectric thickness would be an effective method to deal with 〈*W_d_*〉, while the ferroelectric characteristics remain.

**Figure 10:**
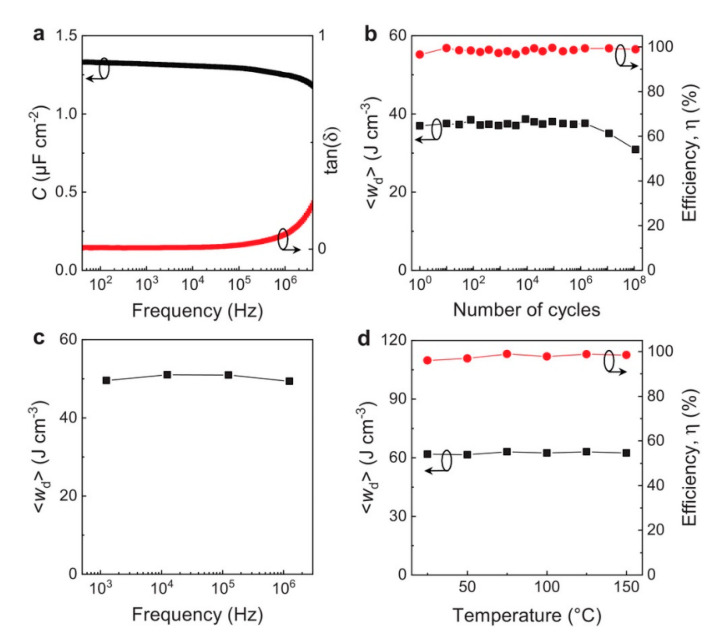
Plots of major factors with the stability of the capacitor composed with ferroelectric/dielectric stack. (**a**) Both capacitance density *C* (left) and loss factor tan (δ) (right) versus frequency without DC bias for frequency stability. (**b**) Discharged energy density 〈*W_d_*〉 (left) and efficiency (right) versus cycling times under 10.6 V for cycling stability. (**c**) 〈*W_d_*〉 versus frequency by pulse under 12.7 V for frequency stability. (**d**) 〈*W_d_*〉 (left) and efficiency (right) versus temperature at 14.4 V for temperature stability. Reprinted with permission from Ref. [16]. Copyright 2019. The authors are under Creative Commons License 4.0 (https://creativecommons.org/licenses/by/4.0/).

The frequency, including the small-signal capacitance per area and loss factor/pulse with 〈*W_d_*〉, temperature, and the cycling stability of the capacitor composed with ferroelectric/dielectric stack are checked in Figure 10. The Tan (δ) measured with 0.6% during *C* is about 1.3 µF cm^−2^. While the cycles are up to 1,000,000 times, the discharged energy density stays at about 38 J cm^−3^ below the voltage of 10.6 V. While the efficiency is maintained at about 99%, 〈*W_d_*〉 decreases to about 31 J cm^−3^ on 100,000,000 times of cycling by the pinning of ferroelectric domains which would not affect the capacitance enhancement. The capacitor composed with HZO layer exhibited high both discharged energy density and efficiency during an increase in temperature of up to 150 °C. These experimental results show that a supercapacitor based on negative capacitance effect operation could be feasible based on the described designs.

**Figure 11:**
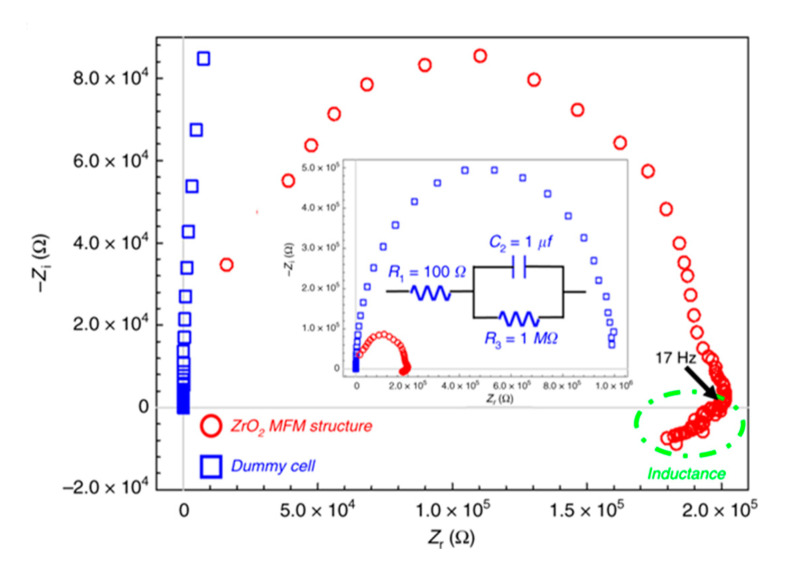
Nyquist plot based on the real and imaginary parts at the complex impedance (Zr + jZi) of MFM capacitor stacked with Pt–ZrO2–Pt. Resistor-capacitor (RC) dummy cell provided by Metrohm AUTOLAB, KM Utrecht, Netherlands is exhibited in an insert with RC component 100 Ω and an RC element (1 μF) capacitance with 1 MΩ resistance integrated in parallel. Reprinted with permission from Ref. [52]. Copyright 2019. The authors are under Creative Commons License 4.0 (https://creativecommons.org/licenses/by/4.0/).

Additionally, the fact-confirming evidence and theoretical perspective for negative capacitance and inductance induced by polarization switching is also discussed in Figure 11. The analysis is described with the plot of the real and imaginary Nyquist impedances of ferroelectric capacitors based on nanoscale zirconium oxide (nano-f-ZrO_2_). Measurements were conducted with an impedance analyzer in the frequency range from 1 MHz to 10 mHz at 1 V direct current (DC) voltage and 10 mV alternating current (AC) perturbation. You can see that the RC circuit (shown in the inset of Figure 11) gives a semi-circle of negative imaginary impedance in the Nyquist plot [52].

## 5. Theories of Pseudocapacitors

In the mid-1920s, scientists discovered the presence of pseudocapacitance in some electrode materials of metal-ion batteries, and they called this phenomenon specific adsorption, which is based on chemical processes [53]. The term “pseudocapacitance” is synonymous with the surface redox reaction, which is based on chemical reactions, such as batteries. When electrical energy is stored in a solid material by chemical reaction, a material with high electrochemical potential is formed.

As the high electrochemical potential of material based on the chemical reaction of a solid substance is mainly stored in chemical bonds which includes ionic bonds, covalent bonds, and hydrogen bond, the material is disconnected or changed with chemical bonds to the low electrochemical potential of the material, the electrochemical reactions emitting chemical energy that can be transferred as electrical energy is generated. The difference in the reaction between the state of charge and state of discharge can be explained by the following equation with the Gibbs free energy:

(15) ΔG=ΔU+PΔV−VΔP−TΔS−SΔT.

The energy transformation of a given chemical reaction can be explained by the Gibbs free energy. In the equation, *G* is the Gibbs free energy, the internal energy of the system is represented as *U*, the pressure is expressed as *P*, the temperature is expressed as *T*, and *V* is the volume. The capacitance of the pseudocapacitor can be 10–100 times higher than that of the EDLCs [20,54,55].

Both pseudocapacitors and batteries are based on chemical reactions, but there are major differences in the charge and discharge behaviors of pseudocapacitive materials in different time domains. Pseudocapacitance, a faradaic activation involving surface or near-surface redox phenomenon, offers a means of achieving high energy density at high charge-discharge rates. Pseudocapacitors are not capacitors with a conventional mechanism perspective of using only electrical energy. However, output properties are shown as capacitors rather than batteries.

**Figure 12:**
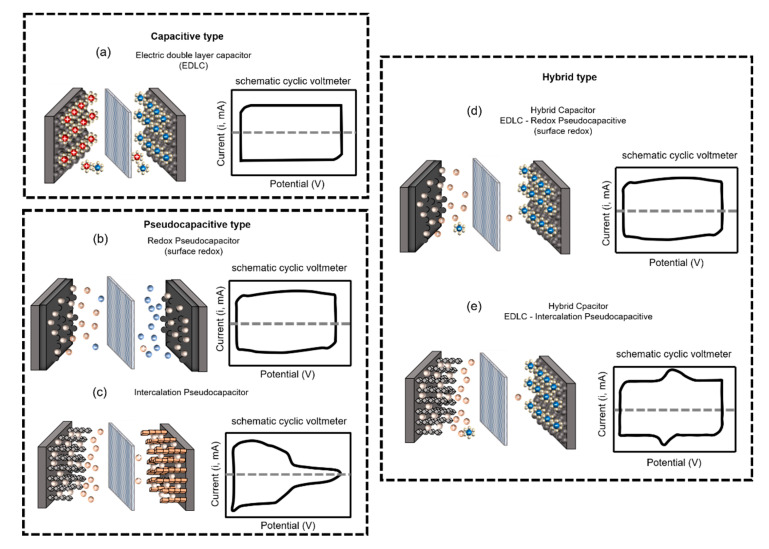
Schematic illustration of a supercapacitor classified by capacitive type. (**a**) Schematic illustration of EDLC, which has cations and anions that are physically layered from electrodes. The schematic cyclic voltmeter has the same shape as a capacitor. (**b**) Schematic illustration of redox pseudocapacitor, which stores and releases energy through chemical adsorption reactions at the surface of the electrode. The schematic cyclic voltmeter is not the same as a capacitor but has a similar shape. (**c**) Schematic illustration of intercalation pseudocapacitor, which stores and releases energy through chemically transform reactions based on the faradaic charge storage mechanism. The schematic cyclic voltmeter shows the characteristics between the capacitor and the battery. (**d**) Schematic illustration of hybrid capacitor integrated both electrode types, which is EDLC and redox pseudocapacitive material. (**e**) Schematic illustration of hybrid capacitor integrated with both electrode types, which are EDLC and intercalation pseudocapacitive material [5,6,10,21,55].

In rare cases, pseudocapacitive materials (lithium in Nb_2_O_5_) have exhibited interlayer insertion processes which are essential phenomena of intercalation pseudocapacitance rather than rapid reactions through conventional surface redox reactions. Since the concept of “intercalation pseudocapacitance” was proposed, it was no longer the synonymous mean of the surface redox reaction. The intercalation pseudocapacitance shows a faradaic charge storage mechanism, which is more similar to batteries than the redox pseudocapacitance. The cyclic voltammetry results of intercalation pseudocapacitance appear closer to that of a battery than a capacitor. Supercapacitors can satisfy their purpose by design, from EDLCs showing typical capacitor characteristics to pseudocapacitors showing a medium performance as capacitors and batteries (Figure 12) [10,56,57].

Pseudocapacitive types are usually based on electrode materials regarding their specific capacitance. There are various materials with their theoretical capacitance listed in Table 3. The composites exhibited their characteristics according to the properties of materials that have identical advances and challenges. CNT and graphene, which demonstrate stability in various regions including chemical, thermal, and mechanical consolidations, could demonstrate a composite showing the intermediate properties of the other materials. Table 4 shows various data, including graphene/MnO2/CNTs, GR/MCNTs/MnO_2_, CNTs/PANI/GR, CNTs/GO/PANI, and CNTs/PANI, which exhibited specific capacitances of 372, 355, 477, 569, 589, and 838, which are significantly enhanced compared to a device manufactured with only graphene and CNTs. Additionally, these composite exhibited life cycling times with significant enhancement, which is considered as the fatal limitation of conducting polymers. 3D Co_3_O_4_/MnO_2_, 3D Co_3_O_4_/MnO_2_, Co_3_O_4_/MnO_2_@GO, Co_3_O_4_/MnO_2_@GO, NiCo_2_O_4_–MnO_2_/GF, Co_3_O_4_, and CuCo_2_O_4_/NiO are demonstrated with a significant capacitance compared to the other composite due to the characteristics of both Co_3_O_4_ and NiO, which have theoretical specific capacitances of 3560 and 2573. However, these composites have fatal drawbacks in both materials, since the composites have not only advantages but also drawbacks. These supercapacitors exhibited fatal limitations on cycling times or chemical, physical, or thermal stabilities. 

Additionally, there are devices taking advantage of properties with specific architectures such as micro flowers, nanosheets, nanobelts, etc. ZnCo_2_O_4_ with Ni foam fabricated in a microflower structure exhibited a superior specific capacitance with a reasonable cycling stability. While CuCo_2_O_4_ with Ni foam consolidated with nanosheets types shows a specific capacitance of 1330 F g^−1^ with 70% capacitance retention after 5000 charge/discharge cycles, CuCo_2_O_4_ fabricated in a nanobelt structure exhibited a 809 specific capacitance with a 127% capacitance retention after 1800 cycles at 2 mA cm^−2^ in Table 5.

Intercalation pseudocapacitance is based on the faradaic charge storage mechanism conventionally used for batteries with high energy densities rather than reversible or near-surface redox reactions. The material with pseudocapacitive characteristics exhibited the electrical response of the EDLC identically. As the charge state changes continuously with the potential, it results in a proportional constant, which is the reason why these devices could be considered as a capacitance.

In pseudocapacitive electrodes, different charge storage mechanisms can be distinguished potential difference deposition, redox reactions of transition metal oxides, intercalation pseudocapacitance, reversible electrochemical doping, and dedoping in conductive polymers. The materials used to fabricate these electrodes are usually metal oxides, mixed metal oxides, sulfides, selenides, nitrides, conductive polymers, carbon materials, and composites with carbon materials. Since materials with various elements and structures may exhibit different electrochemical potentials, it is necessary to carefully design the chemical bonding of solid materials that can be controlled by the surface and interfacial chemical reactions [6,94].

The limitations of the pseudocapacitor are similar to that of the battery, and the degree is also located between that of the EDLC and the battery. As the chemical mechanism of the slow parody process occurs, the performance of specific power by the pseudocapacitor is generally lower than that of the EDLCs. Electrodes exhibiting pseudocapacitance are susceptible to expansion and contraction during charge and discharge cycles, resulting in a poor mechanical stability and shortened cycle life; they are very trivial compared to batteries, which are based on chemical energy with the bulk electrode region and not only the surface layer. However, they show a higher energy storage capacity than that of EDLCs and have both an electrochemically reversible structure to achieve a high cycle compared to a battery and a high power density.

## 6. Other Next-Generation Supercapacitors

### 6.1. Redox Electrolyte Enhanced Supercapacitors

As there are confinement strategies with enhancement on the energy storage capacity with supercapacitors, utilizing various materials could be attractive as electrode materials and structures, which have a higher energy and power than conventional supercapacitors or electrolyte materials. There are different suggestions that are not a conventional method or based on negative capacitance effect but that rely on the redox additive/active electrolyte. Alternative approaches can demonstrate enhancement in various electrode materials; for instance, active carbon electrodes could demonstrate a specific capacitance with 220 F g^−1^, 630.6 F g^−1^, and 901 F g^−1^ at 2.65 mA cm^−2^ and 1 A g^−1^. Active carbon electrolyte devices have redox additive electrolytes such as VOSO_4_ or hydroquinone [95,96,97].

Electrolytes could be divided into three groups. The first group is redox additive-liquid electrolytes. Secondly, there are the redox-active liquid electrolytes. The last group is redox additive-polymer gel electrolytes. Supercapacitors based on redox electrolyte-enhanced supercapacitors could be comparable to batteries. However, there are issues to solve regarding redox electrolyte-enhanced supercapacitors compared to the liquid electrolytes with a low ion mobility and ion accessibility with the electrode, causing a poor contact area [23].

### 6.2. Piezoelectric Supercapacitors

Piezoelectric materials have attracted particular attention to energy harvesting technology by means of nanogenerators. As devices fabricated with zinc oxide nanowire arrays could be bent, strain field and charge separation are formed by the coupling of a piezoelectric layer with zinc oxides which exhibit semiconducting properties. The separator utilized on a supercapacitor could be replaced by a piezoelectric film which transforms physical stress with piezoelectric potential [98]. 

Conventional piezoelectric films that have issues of stability, flexibility, and cost had been proposed with promising alternatives such as polyamide (PA) or polyvinylidene fluoride (PVDF) with an enhanced β crystalline phase content [99]. The piezoelectric layer stressed by the environment generates the potential to drive anions or cations with electrochemical potential. The polarization of ions generates a potential difference with the thickness of the separator. The piezoelectric field induces the movement of H+ to screen piezoelectric field. To obtain chemical equilibrium, oxidation and redox reactions should occur on the positive electrode and negative electrode. Devices operating based on mechanical potential without external electric energy have been demonstrated [100]. A flexible piezoelectric device has been proposed with carbon clothes with a maximum specific capacitance of 357.6 F m^−2^ at 8 Am^−2^ [101]. A PVDF-ZnO film supercapacitor applied a mechanical force charged from 35 to 145 mV with 300 s [102]. 

Additionally, other devices generate electrochemical energy with a different method to that of piezoelectric supercapacitors; they are called thermal self-charging supercapacitors. Thermal self-charging with an electrochemical double layer capacitor in an open circuit with a bias of 80–300 mV is achieved with a thermal energy of 65 °C. The charge generation could be achieved at room temperature after the device is heated at a high temperature.

### 6.3. Electrochromic Supercapacitors

Electrochromic supercapacitors quantifiably determine their electrical capacity by an optical method based on some electrode materials which had demonstrated their color transformation during electrochemical operation [103]. The visual change is the most noticeable thing to figure out. Normalized optical density could be associated with energy density variation, since color according to remaining electrical capacity needs a significant definition. Calibration curves are dependent on various hybrid supercapacitors, which could be a method to measure energy capacity.

A tungsten electrode which changes from transparent to blue is considered the most promising device due to compatibility with both supercapacitors and electrochromic materials [104]. Electrochromic supercapacitors are a method to develop devices with imaginative applications.

### 6.4. Fiber Shaped Supercapacitors

Flexible and portable electronic devices have potential to become mainstream in the Fourth Industrial Revolution, which facilitates the emergence of a hyper-connected society, and it is the basis for data processing between people and things as well as between things and things. The emerging fiber-shaped supercapacitors have helped to create ESSs or portable electronic systems. However, conventional devices have a specific structure based on a planar format. 

These formats had been changed by fabrication with carbon nanotubes (CNTs), which demonstrate high flexibility, tensile strength, electrical conductivity, and stability. However, CNT materials have significantly low specific capacitances, with 4.5 to 5 F g^−1^ [105]. Additionally, there are challenges of strategies with electrochemical performance and physical stability, including flexibilities. The stretchable asymmetric FSS demonstrated a specific capacitance of 60.435 mF cm^−2^ at 10 mV s^−1^. To enhance the energy density, ppy, which demonstrates a broad range of negative potential and MnO_2_, would be electrodeposited onto CNT film (CNT@PPy, CNT@MnO_2_) due to the inherently high energy density of composites. The FSS demonstrated significant stabilities with an 88% capacitance after 200 stretching cycles with physically a 20% strain [106].

### 6.5. Shape Memory Supercapacitors

Shape memory supercapacitors are usually based on shape memory polymer fibers with recoverable materials such as CNT and graphene. These supplement materials reinforce shape memory polymer fibers with electrical conductivity, recovering stability, or reduction in stress. Representative materials of shape memory supercapacitors are usually MWCNT, PANI, and polymer electrolyte, which could be recovered through thermal energy, triggering shape memory effects [107]. MWCNT has unique properties based on a large aspect ratio that could endure huge mechanical stress without noticeable degradation [108]. CNT-coated polyurethane–poly(ε-caprolactone) PU-PCL electrode demonstrated a superior cycle stability, with 96.5% capacitance retention after 10,000 cycles due to the recovering stability of the substrate with poly(acrylic acid)–poly(ethylene oxide), which is used as electrolyte. Additionally, the PU-PCL supercapacitor exhibits a specific capacitance with 37 F g^−1^ at 0.5 A g^−1^ [109]. MWCNT/MnO_2_/SMPU hybrid film not only could be changed with various structures with mechanical forces but also could reconsolidate its initial formation within 1 sec when the thermal environment is adjusted above a glass transition temperature. The device demonstrates a specific capacitance of 69.1 mF cm^−2^ and a cycle stability with a 95.3% capacitance retention after 3000 cycles [110]. These recoverable and structure adjustable supercapacitors are considered as the most prospective devices for applications such as electronic textiles, flexible electronic systems, and wearable devices [109,110].

## 7. Conclusions

As the world which facilitates the emergence of a hyper-connected society requires next-generation ESS, supercapacitors draw attention as one of the candidates with their superior properties in many key indicators, such as power density, cycle stability, temperature range, and safety, except for energy storage capacity, which could be considered as the most important factor in the prevalent view. The major frameworks to solve these issues conventionally focus on utilizing different materials and facial structures. We discuss various electrodes—not only materials such as ACs, MWCNT, metal oxides (MnO_2_, RuO_2_, etc), metal hydroxides (Ni(OH)_2_, Co(OH)_2_, etc.), and conducting polymers (PANI, PPy, etc), but also redox electrolyte. However, it is not enough to satisfy the demand for energy capacity with only these strategies. In this work, we synthesize not only the charge repositioning principle according to supercapacitor types, but also various interaction theories of electrodes with electrolytes which consist of both a dielectric layer and a charge separation layer. We suggest that a novel theory reported recently by a research group focusing on negative capacitance demonstrated the energy storage capacity enhancement through the combination of ferroelectric and dielectric layers could be one of the solutions to the noticeable hurdle that supercapacitors should overcome. Additionally, next-generation supercapacitors, including redox electrolyte-enhanced capacitors, which rely on the enhancement on energy storage capacity with the redox electrolyte; piezoelectric supercapacitors, which could generate energy by means of nanogenerators, electrochromic supercapacitors, which could quantifiably determine their electrical capacity via appearance, and fiber-shaped supercapacitors and shape memory supercapacitors, which are presented for wearable or stretchable energy storage systems, were described regarding the promising potential of supercapacitors.

## Figures and Tables

**Table 1:** Description of the properties of energy storage systems.

Energy Storage System	Capacitors	Supercapacitors	Batteries
**Specific energy density (Wh kg^−1^)**	0.01 to 0.1	0.1 to 50	10 to 200
**Specific power density (W kg^−1^)**	10^3^ to ~10^7^	1 to ~10^6^	10 to 100
**Charge storage mechanism**	Charge Separation	Charge Separation Charge adsorption (desorption) Intercalation (deintercalation)	Faradaic Intercalation (deintercalation)
**Storage region**	Surface	Surface	Surface to bulk
**Cycling performance**	Infinite	>500,000	500~2000
**Charge temperature (°C)**	−20 to 100	−40 to 65	0 to 45
**Discharge temperature (°C)**	−20 to 100	−40 to 65	−20 to 60
**Charging time (sec)**	10^−6^ to 10^−3^	1 to 10	10^3^ to 10^5^
**Galvanostatic discharge curves**	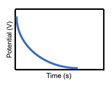	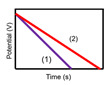 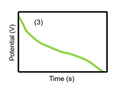	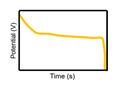

Note: Galvanostatic discharge curves of supercapacitors were classified into three types: (1) EDLC, (2) Pseudocapacitors (redox), and (3) Pseudocapacitors (intercalation).

**Table 2:** Properties of various electrode materials [21,22,23].

Electrode Materials	Advantages	Issues and Challenges	Electrode Description
**Activated carbons**	high surface area (over 1000 m^2^ g^−1^) low cost, chemical stability, and availability	specific capacitance, conductivity	AC
**CNT/Graphene**	high surface area (over 2600 m^2^ g^−1^), thermal conductivity, flexibility, chemical, thermal, mechanical stability corrosion resistance	volumetric capacitance, agglomeration, nano–micro transformation,	GO MWCNT
**Metal oxides-** **pseudocapacitance**	high specific capacitance, porosity, adhesion, conductivity	ion accessibility, intrinsic stability, relatively high cost	MnO_2_, RuO_2_
**Metal oxides/hydroxides-faradaic**	high specific capacitance, long cycles life, high conductivity, good electrochemical reversibility, and high rate capability	stability, barren reveres, relatively high cost	Co_3_O_4,_ NiO, CuO, Fe_2_O_3,_ TiO_2_, etc. Metal hydroxides (Ni(OH)_2_, Co(OH)_2_)_,_
**Conducting polymers**	relatively high storage capacity, low cost, low environmental impact, porosity high voltage window,	conductivity, stability	PANI, PPy, PTh

**Table 3:** The theoretical capacitance of various composite with issues.

Composite	Specific Capacitance (F g^−1^)	Issue
NiO	2573	Cycling stability
Co_3_O_4_	3560	Toxicity, Cycling stability
CuCo_2_O_4_	984	Cycling stability
V_2_O_5_	2120	Low conductivity, Cycling stability
MnO_2_	1380	Limited thickness layer
RuO_2_, xH_2_O	1200–2200	High cost, blockage of accessible surface area

**Table 4:** Specific capacitance of various composites.

Composite	Specific Capacitance (F g^−1^)	Electrolyte Solution	Current Density (*i_c_*)/Scan Rate (*v*)	Year	Ref.
MnO2/porous carbon	459	KOH	1.0 A g^−1^	2014	[58]
GO/MnO2	315	1 m Na_2_SO_4_	0.5 A g^−1^	2015	[59]
3D GR/MnO2	267	1.5 M Li_2_SO_4_	200 mV s^−1^	2018	[60]
3D Co3O4/MnO2	1397	2.0 M KOH	1 mA cm^−2^	2016	[61]
3D Co3O4/MnO2	1184	1 M LiPF_6_	1.0 A g^−1^	2014	[62]
MnO2@GO	1518	1.0 M Na_2_SO_4_	1.0 A g^−1^	2020	[63]
Co3O4/MnO2@GO	1358	1.0 M Na_2_SO_4_	1.0 A g^−1^	2020	[63]
Co3O4/MnO2@GO	1718	1.0 M Na_2_SO_4_	1.0 A g^−1^	2020	[63]
graphene/MnO2/CNTs	372	1 m Na_2_SO_4_	0.5 A g^−1^	2012	[64]
GR/MCNTs/MnO2	355	1 m Na_2_SO_4_	0.3 A g^−1^	2013	[65]
CNT/TiO2/PANI	477	0.5 M H_2_SO_4_	0.4 μA mm^−2^	2013	[66]
CNTs/PANI/GR	569	1.0 M HCl	0.1 A g^−1^	2011	[67]
CNTs/GO/PANI	589	1.0 M H_2_SO_4_	0.2 A g^−1^	2013	[68]
CNTs/PANI	838	1.0 M H_2_SO_4_	1 mV s^−1^	2011	[69]
MWCNTs/PANI	233	H_3_PO_4_-PVA gel	1 A g^−1^	2013	[70]
MWCNTs/PPy	427	1.0 M Na_2_SO_4_	5 mV s^−1^	2010	[71]
MWCNTs/PPy/MnO_2_	365	0.5 M Na_2_SO_4_	5 mV s^−1^	2014	[72]
MWCNTs/PANI	560	0.1 M H_2_SO_4_	1 mV s^−1^	2010	[73]
MWCNTs/Pd/PANI	920	1.0 M H_2_SO_4_	2 mV s^−1^	2013	[74]
d-CNTs/PPy	587	0.1 M NaClO_4_	3 A g^−1^	2012	[75]
C60-PANI-EB	776	1.0 M H_2_SO_4_	1 mA cm^−2^	2012	[76]
MnO2/AC	324	Na_2_SO_4_	0.1 A g^−1^	2008	[77]
MnO2/AC	345	KOH	10 mA cm^−2^	2015	[78]
δ-MnO2/AC	360.5	1M NaNO_3_	4 A g^−1^	2020	[79]
MnO2/CNT	199	1.0 M Na_2_SO_4_	0.1 A g^−1^	2008	[80]
MnO2/CNT	325.5	1 m Na_2_SO_4_	0.3 A g^−1^	2009	[81]
NiCo2O4–MnO2/GF	2577	1 m Na_2_SO_4_	1 A g^−1^	2017	[82]
PPy-graphene	165	1.0 M NaCl	1 A g^−1^	2010	[83]
PAA@MnO2/PPy	564	0.2 M FeCl_3_	10 mV s^−1^	2018	[84]
PAA@MnO2/PPy	692	0.2 M FeCl_3_	0.5 A/g	2018	[84]
PAA@ MnO2	288	0.2 M FeCl_3_	10 mV s^−1^	2018	[84]
PANI-Si	409	0.5 M H2SO_4_	40 mA cm^−2^	2010	[85]
PANI/MnO_2_	1292	1.0 M LiClO_4_	4.0 mA cm^−2^	2010	[86]
MWCNTs/MnO2/PPy	806	1 M Na_2_SO_4_	1 A g^−1^	2019	[87]
NiO	1700	6 M Hg/HgO KOH	2 A g^−1^	2012	[88]
Co_3_O_4_	2735 to 1471	2 M KOH	2 to 10 A g^−1^	2012	[89]
CuCo2O4/NiO	2219	1.0 M NaOH	1 A g^−1^	2017	[90]
CuCo2O4	743	1.0 M NaOH	1 A g^−1^	2017	[90]
NiO	1296	1.0 M NaOH	1 A g^−1^	2017	[90]

Note: Polypyrrole (PPy), Polyaniline (PANI), Polyacrylic acid (PAA), GR (graphene), GO (graphene oxide), MCNTs (carbon nanotubes), MWCNT (Multi-walled carbon nanotube), and CNT (carbon nanotube).

**Table 5:** Composite with specific architectures with various factors.

Composite	Specific Capacitance (F g^−1^)	Cycling Stability	Electrolyte Solution	Current Density (*i_c_*)/Scan Rate (*v*)	Year	Ref.
ZnCo_2_O_4_-Ni foam (microflowers)	2256	90% capacitance retention after 2000 cycles at 10 mA cm^−2^	1 M KOH	2 mA cm^−2^	2017	[91]
ZnCo_2_O_4_-Ni foam (microflowers)	1700		1 M KOH	30 mA cm^−2^	2017	[91]
ZnCo_2_O_4_-Ni foam (nanosheets)	2037	80% capacitance retention after 2000 cycles at 10 mA cm^−2^	1 M KOH	2 mA cm^−2^	2017	[91]
ZnCo_2_O_4_-Ni foam (nanosheets)	719		1 M KOH	30 mA cm^−2^	2017	[91]
CuCo_2_O_4_ (nanobelts)	809	127% capacitance retention after 1800 cycles at 2 mA cm^−2^	2.0 M KOH	0.667A g^−1^	2015	[92]
CuCo_2_O_4_-Ni foam (nanosheets)	1330	70% capacitance retention after 5000 cycles at 2 A g^−1^	3 M KOH	2 A g^−1^	2018	[93]
CuCo_2_O_4_-Ni foam (nanosheets)	938		3 M KOH	60 A g^−1^	2018	[93]
